# Cross-Calibration between ASTER and MODIS Visible to Near-Infrared Bands for Improvement of ASTER Radiometric Calibration

**DOI:** 10.3390/s17081793

**Published:** 2017-08-04

**Authors:** Kenta Obata, Satoshi Tsuchida, Hirokazu Yamamoto, Kurtis Thome

**Affiliations:** 1 National Institute of Advanced Industrial Science and Technology (AIST), The Institute of Geology and Geoinformation, Tsukuba, Ibaraki 305-8567, Japan; s.tsuchida@aist.go.jp (S.T.); hirokazu.yamamoto@aist.go.jp (H.Y.); 2 NASA Goddard Space Flight Center, Greenbelt, MD 20771, USA; kurtis.thome@nasa.gov

**Keywords:** ASTER, MODIS, VNIR, cross-calibration, RCC, radiance, uncertainty

## Abstract

Radiometric cross-calibration between the Advanced Spaceborne Thermal Emission and Reflection Radiometer (ASTER) and the Terra-Moderate Resolution Imaging Spectroradiometer (MODIS) has been partially used to derive the ASTER radiometric calibration coefficient (RCC) curve as a function of date on visible to near-infrared bands. However, cross-calibration is not sufficiently accurate, since the effects of the differences in the sensor’s spectral and spatial responses are not fully mitigated. The present study attempts to evaluate radiometric consistency across two sensors using an improved cross-calibration algorithm to address the spectral and spatial effects and derive cross-calibration-based RCCs, which increases the ASTER calibration accuracy. Overall, radiances measured with ASTER bands 1 and 2 are on averages 3.9% and 3.6% greater than the ones measured on the same scene with their MODIS counterparts and ASTER band 3N (nadir) is 0.6% smaller than its MODIS counterpart in current radiance/reflectance products. The percentage root mean squared errors (%RMSEs) between the radiances of two sensors are 3.7, 4.2, and 2.3 for ASTER band 1, 2, and 3N, respectively, which are slightly greater or smaller than the required ASTER radiometric calibration accuracy (4%). The uncertainty of the cross-calibration is analyzed by elaborating the error budget table to evaluate the International System of Units (SI)-traceability of the results. The use of the derived RCCs will allow further reduction of errors in ASTER radiometric calibration and subsequently improve interoperability across sensors for synergistic applications.

## 1. Introduction

The Advanced Spaceborne Thermal Emission and Reflection Radiometer (ASTER) flying on the Terra, the flagship of the Earth Observing System (EOS), was launched in 1999 and has now observed the Earth for more than 17 years. The Advanced Spaceborne Thermal Emission and Reflection Radiometer consists of a visible-to-near-infrared (VNIR) subsystem, a shortwave infrared (SWIR) subsystem, and a thermal infrared (TIR) subsystem [1]. The VNIR subsystem consists of two telescopes that look nadir and backward, respectively. The nadir-looking telescope measures three bands with a resolution of 15 m, and the backward-looking telescope measures the NIR band with a resolution of 15 m. The data of NIR bands from the two telescopes are used to create stereo images in order to construct a digital elevation model (DEM). The SWIR subsystem measures six bands with a resolution of 30 m, and the TIR subsystem measures five bands with a resolution of 90 m. Use of the data from the SWIR subsystem obtained since May 2008 are not recommended due to the rise in temperature of the detectors, which has resulted in saturation and severe striping [2].

The radiometric calibration of ASTER VNIR bands for nadir-looking, which is the focus of the present study, has been conducted in order to provide reliable information of radiometric measurements of the sensor in pre-launch and in-orbit periods. The three bands were calibrated to be traceable to the copper fixed-point blackbody as a primary standard, and the calibration was transferred to onboard calibration lamps and their monitoring photodiodes [3,4]. After launch, electric and optical devices of the sensor are degraded with time by several factors associated with the harsh space environment (e.g., high-energy solar radiation and outgassing), which significantly decrease the accuracy of the radiometric measurements. The degradation of each band was monitored by the onboard lamp calibration unit every 17 days during the period of initial check-out and is currently monitored every 49 days [5]. Vicarious calibration using the reflectance-based method has been conducted by Saga University, the University of Arizona, and the National Institute of Advanced Industrial Science and Technology (AIST) a few times a year since launch [6]. Cross-calibration was conducted as a supplemental calibration method [7] using data from the highly calibrated Terra-Moderate Resolution Imaging Spectroradiometer (MODIS), which is mounted on the same platform as ASTER, that is the International System of Units (SI)-traceable through pre-flight calibration [8]. At present, the degradation of ASTER bands 1 and 2 is corrected through a combination of vicarious calibration and cross-calibration, while ASTER band 3N degradation is corrected primarily by onboard calibration [9,10]. The latest version of the radiometric calibration table, referred to as a radiometric database (DB), for VNIR bands is version 4 [10], which contains not only coefficients for radiometric degradation but also gain factors for each band and the sensitivity and offset of each detector, among other information [11]. The method of cross-calibration for the degradation correction is, however, not sufficiently matured, since the effects of differences in spectral registration and spatial resolution across sensors are not fully taken into accounted. Moreover, data used in the cross-calibration are based on a previous version of the radiometric DB, the gain factors for the electrical circuit of which are not identical to those of the latest version, as reported in the onboard electrical calibration [5].

Numerous studies have reported the cross-calibration of ASTER and Terra-MODIS VNIR bands (comparisons between ASTER and MODIS radiometric measurements over time) [7,12,13,14]. Among these studies, trends in the cross-calibration results are not necessarily consistent. One reason for this is the differences between the versions of the radiometric DBs used in the ASTER radiometric calibration. The latest study for the cross-calibration between ASTER and MODIS VNIR bands over the period from 2000 to 2010 [14] used the current radiometric DB, which is more appropriate for discussing the cross-calibration results. In the cross-calibration, the effects of differences in relative spectral response (RSR) have been overcome by using the spectral band adjustment technique (e.g., spectral band adjustment factor (SBAF)) based on hyperspectral data from Earth Observing (EO)-1 Hyperion [14]. Spectral band adjustment is frequently used in the cross-calibration of satellite sensors [15,16,17,18,19,20,21,22,23] and can be performed using not only Hyperion data but also data obtained by SCHIAMACHY, onboard the European environmental satellite (ENVISAT) [24], and through simulations using atmospheric radiative transfer codes [18,25]. Furthermore, the spatial sensitivity characteristics of MODIS (e.g., point spread function) should be considered in aggregating ASTER data because it impacts cross-calibration results [26]. Cross-calibration between ASTER and MODIS that simultaneously addresses spectral and spatial effects has yet to be reported.

The objective of the present study is to perform radiometric cross-calibration between ASTER and MODIS VNIR bands in order to evaluate the radiometric consistency and derive the radiometric calibration coefficients (RCCs), which are used to derive RCC curves with respect to date in order to correct time-dependent radiometric degradation, based on the cross-calibration with the goal of improving the accuracy of the ASTER radiometric calibration. The present study uses coincident and collocated data of ASTER and MODIS spanning 17 years between 2000 and 2016 for the Railroad Valley Playa in Nevada, USA and adopts an algorithm that compensates for the differences in the spectral and spatial characteristics of sensors. The MODIS data are spectrally adjusted to be compatible with corresponding ASTER bands, and ASTER data are spatially aggregated with a weighting function specific to a single pixel of MODIS 1 km-resolution data. The sensitivity analysis is conducted in order to clarify the uncertainty of the cross-calibration results and the derived RCCs. The derived RCCs are compared to RCCs based on onboard lamp calibration and vicarious calibration using a reflectance-based method.

## 2. Materials and Methods

### 2.1. Site Information

The selection of a site for radiometric calibration (e.g., vicarious calibration using the reflectance-based method and cross-calibration) relies on the characteristics of the surface and climate conditions [27]. The site should exhibit high reflectance (>0.3) in the target band and a high altitude is desirable in order to reduce the impact of atmospheric scattering and transmittance. The surface should be spatially homogeneous and sufficiently extensive to mitigate the impact of geolocation errors and the adjacency effect. A near-Lambertian surface would reduce the effect of the bidirectional reflectance distribution function (BRDF). The temporal variability of the surface spectral properties, including the shape of the spectral reflectance and the BRDF, should be minimal. Sites with little to no vegetation exhibit such high reflectance, low BRDF effects, and temporal and spectral uniformities. A site over an area with a dry climate would be preferable in order to reduce the effects of changes in surface properties due to rain and cloudy weather.

The Railroad Valley Playa located in central Nevada, USA satisfactorily satisfies the above requirements for radiometric cross-calibration [12]. The size of the playa is approximately 15 km by 15 km, with an elevation of 1.5 km, and the playa consists of compacted clay-rich lacustrine deposits that form a smooth surface [25]. The region of interest (ROI) is rectangular (1 km by 2 km for the along- and across-track directions, respectively), and coordinates ($l_{0},\;\;b_{0}$) for the center of the region are N $38.50486^{\circ }\;$/W $-115.69041^{\circ }$. The region around the center of the ROI is used in the vicarious calibration of the ASTER VNIR and SWIR bands using a reflectance-based method, such that we have a data record of surface reflectances for the spectral band adjustment. The BRDF effect might have less of an influence on the cross-calibration because the present study uses almost simultaneous observations from ASTER and MODIS flying on the same platform.

The number of ASTER observations of the Earth is limited due to the fact that ASTER needs to be tasked for observation. However, the Railroad Valley Playa has been observed relatively frequently because of ASTER field campaigns for vicarious calibration, and the number of observations is greater than for other candidate cross-calibration sites.

### 2.2. Satellite Data for Cross-Calibration

ASTER value-added (ASTER-VA) data, which include radiometrically corrected and ortho-rectified radiance products and DEM data, are used in the present study. The National Institute of Advanced Industrial Science and Technology started to distribute ASTER-VA data in April 2016 [28] after Japan Space Systems (JSS) quit distributing similar products, called ASTER Level 3A (L3A). The ASTER-VA data are produced using ASTER Level 1A (L1A) data based on almost the same algorithm as was used to produce ASTER L3A (the algorithm for ortho-rectification is slightly different.) The resampling and projection methods are geographic projection (uniform latitude/longitude) and cubic convolution, respectively. The U.S. Geological Survey and the National Aeronautics and Space Administration (NASA) provides the ASTER Level 1 Precision Terrain Corrected Registered At-Sensor Radiance (ASTER L1T) data, which are constructed by wrapping Landsat functionality in a version of the existing ASTER Level 1B algorithm [29]. The geometric algorithm of ASTER-VA is therefore different from that of ASTER L1T. The most recently available radiometric DB has been used to produce ASTER-VA, L3A, and L1T.

The ASTER data over the ROI are then visually inspected in order to screen anomalous data, in which clouds or cloud shadows overlap the ROI or adjacency clouds are found in the vicinity of the ROI. The radiances of ASTER bands 1 and 2 are sometimes saturated when the observation is conducted in high-gain mode. Any ASTER data that involve saturated pixels over the ROI are excluded from the sample data for cross-calibration.

MODIS Calibrated Radiances, Daily L1B Swath 1 km data (MOD021KM) of Collection 6 were obtained from the Level-1 and Atmosphere Archive and Distribution System (LAADS) web [30], which are radiometrically and geometrically corrected to provide at-aperture reflectances. These data include those for bands 1 through 7, each aggregated to 1 km resolution [31]. MODIS Geolocation Fields Daily L1A Swath 1 km (MOD03) of Collection 6 data were obtained from the same website, which contains geodetic coordinates, ground elevation, solar and satellite zenith, and azimuth angles for each 1 km pixel. The angular information was used in the implementation of the radiative transfer code in the cross-calibration.

### 2.3. Spectral Band Adjustment of MODIS Data for Cross-Calibration

**Figure 1:**
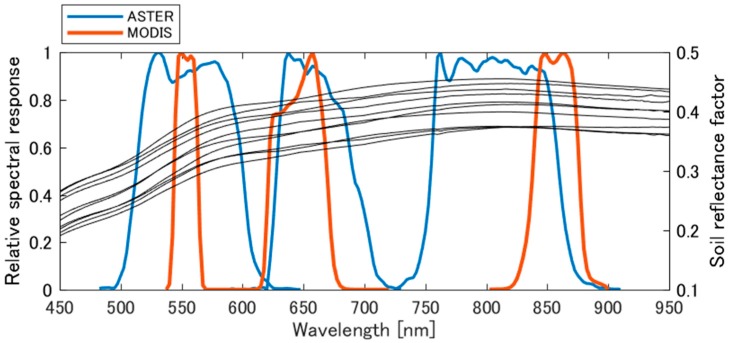
Relative spectral response (RSR) of ASTER bands 1, 2, and 3N and MODIS bands 4, 1, and 2, respectively. Ten samples of soil reflectance factors of the Railroad Valley Playa used in the numerical experiments are plotted.

The effects of RSR between ASTER and MODIS VNIR bands should be mitigated for radiometric cross-calibration. The registration of spectral bands for the sensors is shown in Figure 1. Ten soil reflectance samples of the Railroad Valley Playa, measured by the FieldSpec spectroradiometer, are shown in the figure. The RSRs of ASTER VNIR bands are wider than those of MODIS, and the center wavelength of ASTER band 1 is similar with MODIS band 4. The center wavelength of ASTER band 2 is greater than that of MODIS band 1, whereas that of ASTER band 3N is much smaller than that of MODIS band 2. The differences cause biases between measurements of top-of-atmosphere (TOA) radiances/reflectances of spectral matching bands that stem from the spectral dependency of surface reflectances, atmospheric transmissions, scattering, spherical albedo, etc.

The spectral band adjustment was conducted between corresponding ASTER and MODIS bands. The adjustment involves an inversion of MODIS TOA reflectances using a radiative transfer code, spectral band conversion of surface reflectances of MODIS into those of the ASTER spectral matching band using a linear relationship between the reflectances of the bands over bare soil, i.e., soil line equations [32] and direct computation of ASTER TOA radiances using the radiative transfer code with the input of the converted surface reflectances of ASTER. The historical data of hyperspectral reflectances of the surface are required for deriving soil line equations. Similar approaches for the spectral band adjustment were adopted in cross-calibration studies [33,34].

The calibrated Terra MODIS reflectances are used as a reference because the uncertainty of the calibration is low (2% in reflectance unit [35]) and the sensor is mounted on the same platform as ASTER. The inversion of MODIS TOA reflectances and direct computation of ASTER TOA radiances are conducted using the Second Simulation of a Satellite Signal in the Solar Spectrum, Vector (6SV) 2.1 [36,37] with in-situ measurements of atmospheric conditions for the Railroad Valley Playa. Effects of variations in the atmospheric condition on the spectral band adjustment are expected to be small, because errors in the inversion can be compensated to some extent in the reverse direction [33]. These errors are also identified in the sensitivity analysis introduced in Section 4. However, we use near real-time measurements of the atmospheric condition as far as possible in order for the cross-calibration to be accurate. Note that the solar irradiance model used in 6SV is altered to that distributed by the MODIS Characterization Support Team (MCST), since MODIS is the reference sensor in the cross-calibration. The solar irradiance data from MCST were compiled from the results of Thuillier et al. (400–800 nm) [38], Neckel and Labs (800–1100 nm) [39], and Smith and Gottlieb (above 1100 nm) [40] and were interpolated to 2.5 nm for the wavelength region 250–2397.5 nm. The Junge power-law distribution was selected for the aerosol model.

The input variables of the 6SV code, i.e., the aerosol optical thickness (AOT) at 550 nm, the Junge parameter, the column water vapor, and the column ozone amount, were assumed to change partially every scene. The first three variables were obtained near-simultaneously (within 10 min) upon satellite overpass by the CIMEL 318 automatic tracking sun and sky-scanning radiometer of the AERONET facility installed in the Railroad Valley Playa. The CIMEL 318 basically measures the atmosphere separately at 340, 380, 440, 500, 675, 870, 940, and 1020 nm [41]. The AOT at 550 nm was calculated using the Angstrom parameter based on the AERONET measurements:

(1) $$\tau_{550}=\tau_{500}{\left(\frac{550}{500}\right)}^{-\alpha }$$

where τ and its subscript are AOT and wavelength (nm), respectively, and α is the Angstrom exponent. The Junge parameter γ is approximated as follows:

(2) γ≈α+2

Level 2.0 quality-assured data were used to obtain column water vapor, the AOT at 550 nm, and the Junge parameter. The column ozone amount was obtained through the Goddard Earth Science Data and Information Services Center (GES DISC) [42], and Level-2 ozone total column data were obtained using the Earth Probe-Total Ozone Mapping Sensor (TOMS) and the Aura-Ozone Monitoring Instrument (OMI), the spatial resolutions of which are 50 km × 50 km and 13 km × 24 km, respectively. Level-3 data of TOMS (1.25-degree longitudinal resolution and 1.0-degree latitudinal resolution) were used over the period from September 2003 to September 2004, during which the Level-2 data are missing. Notice that the equator crossing times for the Earth-Probe and Aura are approximately 11:16 a.m. [43] and 1:45 p.m. [44], respectively, and are not coincident with that of Terra (10:30 a.m.) [45]. If the measurements of the parameters on the date are not available, a common atmospheric condition is applied as the input, which is the median of the measurements on multiple dates for cross-calibration based on each of AERONET from 16 July 2001 to 24 August 2015 and the TOMS/OMI from 6 April 2000 to 26 August 2016. The systematic errors caused by using the common atmospheric condition in spectral band adjustment is evaluated in Section 4. In the present study, constant values are used for the real (1.44) and imaginary (0.005) parts of the refractive index [46].

### 2.4. Spatial Registration and Aggregation of ASTER Data for Cross-Calibration

**Figure 2:**
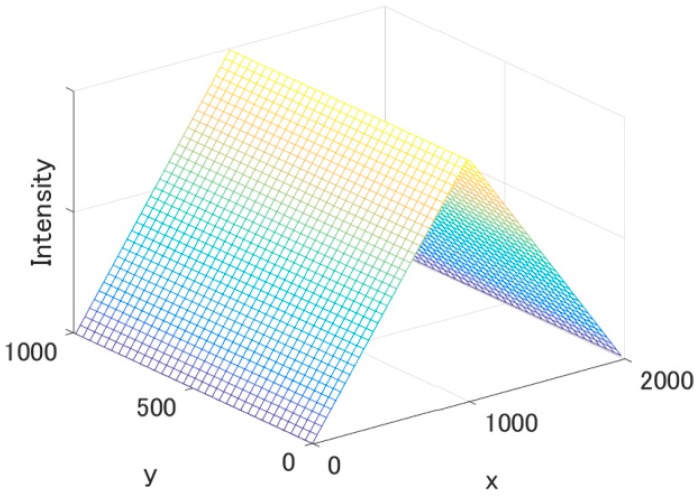
Schematic diagram of the weighting function for convolving ASTER data to be spatially compatible to MODIS aggregated 1 km-resolution data. The units of the x and y axes are meters.

ASTER data are aggregated to simulate the spatial response of the MODIS instantaneous field of view (IFOV) and the aggregation for providing 1 km-resolution data. The IFOV at nadir for 250 m (bands 1 and 2) and 500 m (e.g., band 4) resolutions can be approximated by rectangular and triangular functions for the along- and cross-track directions, respectively [47]. The MODIS 1 km-resolution data are produced by aggregating 28 (4 × 7) and 6 (2 × 3) pixels of 250 m and 500 m resolutions, respectively, with the weighting functions that form the rectangular function for the along-track direction and the triangular function for the cross-track direction. Note that the actual region sensed for the resolution is 1 km (along-track direction) by 2 km (cross-track direction), since only the actual length sensed in the cross-track direction for each pixel is twice the spatial resolution. The ASTER data are then convolved using the weighting functions shown in Figure 2.

First, the MODIS pixel that is closest to the latitude and longitude for the center of ROI, ($b_{0}$, $l_{0}$), is extracted as the sample pixel data, and the coordinates of the pixel are identified by ($b_{m,i}$, $l_{m,i}$), where the subscript *m* indicates MODIS and the subscript *i* identifies an individual date. The Universal Transverse Mercator (UTM) coordinates for the point are also computed and denoted by ($x_{m,i},\;y_{m,i}$). ASTER data on the geodetic coordinates are re-projected into the coordinates of the UTM. A pixel of ASTER data for the i-th date that is closest to ($x_{m,i},\;y_{m,i}$) is extracted and identified as ($x_{a,i},\;y_{a,i}$), where subscript a indicates ASTER. The 1 km × 2 km area surrounding ($x_{a,i},\;y_{a,i}$) is extracted, and the data are convolved with the simulated weighting function shown in Figure 2 to produce ASTER data that are spatially compatible with MODIS aggregated 1 km-resolution data.

### 2.5. Cross-Calibration

The spatially aggregated ASTER radiances are compared with MODIS radiances that are spectrally adjusted to the corresponding ASTER bands. The average of percentage differences between the radiances for each band ($\epsilon_{b}$) is computed as the bias, where the subscript b identifies the band. The root mean square errors between these radiances divided by the mean of the MODIS radiances (${\%\mathrm{RMSE}}_{b}$) is computed as:

(3) $$\epsilon_{b}=\frac{1}{N_{b}}\sum_{i=1}^{N_{b}}\left(\frac{{\bar{L}}_{a,b,i}-{\hat{L}}_{m,b,i}}{{\hat{L}}_{m,b,i}}\right)\times 100$$

(4) $${\%\mathrm{RMSE}}_{b}=\frac{\sqrt{\frac{1}{N_{b}}\sum_{i=1}^{N_{b}}{\left({\bar{L}}_{a,b,i}-{\hat{L}}_{m,b,i}\right)}^{2}}}{\frac{1}{N_{b}}\sum_{i=1}^{N_{b}}{\hat{L}}_{m,b,i}}\times 100$$

where ${\hat{L}}_{m,b,i}$ are spectrally adjusted MODIS radiances, ${\bar{L}}_{a,b,i}$ are spatially aggregated ASTER radiances, and $N_{b}$ is the band-dependent number of sample data.

The temporal trend of the cross-sensor radiometric characteristics is investigated using a scatter plot of the relative differences between ${\bar{L}}_{a,b,i}$ and ${\hat{L}}_{m,b,i}$ vs. date. The periods of the first six years, the next six years, and later periods are denoted as periods 1, 2, and 3, respectively, in order to clarify the statistical characteristics of the relationships between the differences and the date specific to the period. Moreover, $\epsilon_{b}$ and ${\%\mathrm{RMSE}}_{b}$ for each period are also computed. The statistics of the regression line between the differences and the date is computed, including the slope, F-statistics, and *p*-value. The slope of the line is not statistically significant at the 5% level if the *p*-value is greater than 0.05. A statistically significant slope indicates clear trend and that the trend of the scatter plot is not flat, i.e., not stable.

## 3. Results

### 3.1. Atmospheric Parameters and Soil Line Slope and Offset Used in Spectral Band Adjustment

The atmospheric parameters to be changed as the input of 6SV code were obtained, and the statistics of the parameters are shown in Table 1. The number of coincident and collocated observations of ASTER and MODIS for the clear-sky condition after visual screening was 75. The existence of level 2.0 quality-assured data from AERONET and data from TOMS/OMI for the Railroad Valley Playa for the 75 dates was investigated. Measurements of 38 dates were obtained from AERONET and measurements of 74 dates were obtained from TOMS/OMI sensors. The median values for these data, regarded as the common atmospheric condition, are listed in Table 1.

**Figure 3:**
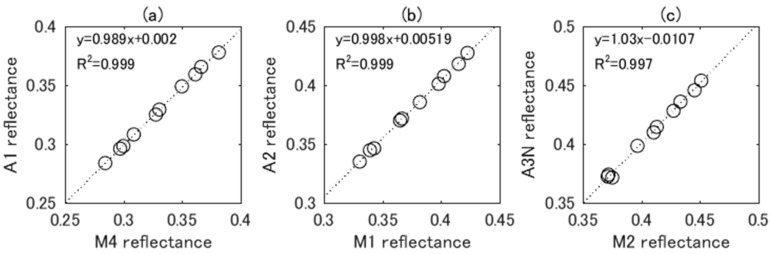
Scatter plots of soil reflectances of the Railroad Valley Playa over ASTER-MODIS reflectance space. (**a**) ASTER band 1 vs. MODIS band 4; (**b**) ASTER band 2 vs. MODIS band 1; (**c**) ASTER band 3N vs. MODIS band 2.

The soil line parameters for ASTER and MODIS spectral matching bands were computed using historical data of the surface reflectance of the Railroad Valley Playa with a 1 nm interval between 350 nm and 2500 nm. Ten samples of the spectrum obtained on different dates are shown in Figure 1, each of which is actually an average of 900 measurements over a 90 m × 80 m area [9]. The spectral reflectances of the 10 samples were spectrally convolved using ASTER and MODIS RSR in order to calculate ASTER band 1, 2, and 3N and MODIS band 4, 1, and 2 reflectances. In the figure, A1, A2, and A3N represent ASTER bands 1, 2, and 3N, and M4, M1, and M2 represent MODIS bands 4, 1, and 2, respectively. Scatter plots of the surface reflectances of the ASTER and MODIS counterparts are shown in Figure 3. The dotted line is the soil line, which was computed by the linear regression. Interestingly, the soil line slope and offset were close to unity and zero, respectively, for each pair of bands.

### 3.2. Cross-Calibration

The total numbers of coincident and collocated data of ASTER and MODIS after the visual screening and removal of data that were affected by the saturated pixels were 44, 58, and 75, respectively, for the green (ASTER band 1 and MODIS band 4), red (ASTER band 2 and MODIS band 1), and NIR (ASTER band 3N and MODIS band 2) portions. Table 2 shows $\epsilon_{b}$ and ${\%\mathrm{RMSE}}_{b}$ for the entire period. As shown in the table, ASTER band 1 is 3.92% greater than MODIS band 4. Moreover, ${\%\mathrm{RMSE}}_{b}$ for the bands was 3.65. ASTER band 2 is 3.64% greater than MODIS band 1. This wavelength region had values similar to those of the green band. Here, ${\%\mathrm{RMSE}}_{b}$ was 4.20, which is slightly greater than the value for the green band. The value of $\;\epsilon_{b}$ for the NIR band was −0.61, which indicates that MODIS band 2 is slightly greater than ASTER band 3N. Moreover, ${\%\mathrm{RMSE}}_{b}$ was 2.32, which is much smaller than for other wavelength regions.

**Figure 4:**
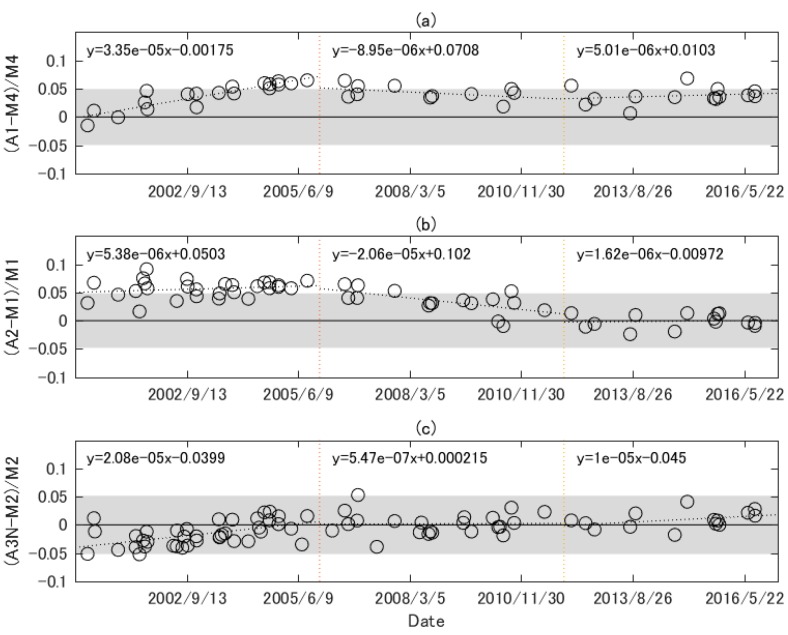
Relative differences between spatially aggregated ASTER radiances for bands 1, 2, and 3N and spectrally adjusted MODIS radiances for bands 4, 1, and 2: (**a**) ASTER band 1 and MODIS band 4; (**b**) ASTER band 2 and MODIS band 1; (**c**) ASTER band 3N and MODIS band 2. The shaded areas indicate the range inside the estimated uncertainty in ASTER-MODIS cross-calibration using calibrated ASTER and MODIS data.

Figure 4a shows the relative differences between spatially aggregated ASTER band 1 radiances and spectrally adjusted MODIS band 4 radiances as a function of date. The entire period is divided by vertical dotted lines in order to distinguish the three periods in the figure. The first few points were close to unity in period 1, but later points gradually shifted upward. The slope of the linear regression line was statistically significant (*p*-value < 0.05, as shown in Table 3), and thus the trend is not stable. The statistically significant slope of the line may be attributed to the time-dependent biases in the radiometric calibration of sensors, although the source is uncertain. The source of these biases may be biases included in ASTER vicarious calibration (e.g., biases in the calibration of the white reference panel), biases in the gain factors of each band, RSR relative shift between ASTER and MODIS, and biases in the MODIS reflectance calibration. The points in period 2 slightly decreased with time, and period 3 was flatter. The slopes of the regression lines were not statistically significant, indicating the stability of the trend. For this result, $\epsilon_{b}$ was 3.7 to 4.3, and ${\%\mathrm{RMSE}}_{b}$ was 3.6 to 3.7. The shaded area indicates the range of estimated uncertainty of the cross-calibration calculated in Section 4, and some points exceeded the range of estimated uncertainty, indicating that additional errors might be unaccounted for in the estimation of the uncertainty and/or the individual sources of errors may have been underestimated.

Figure 4b shows the relative differences between spatially aggregated ASTER band 2 radiances and spectrally adjusted MODIS band 2 radiances as a function of time. The plot exhibits a flatter trend in period 1, which shifted upward by more than 5% ($\epsilon_{b}$ was 5.65). For period 2, the plot was a decreasing function of time, and the slope was statistically significant, perhaps due to the time-dependent biases mentioned previously. After period 2, the trend was stable, and $\epsilon_{b}$ was very close to zero. Good agreement with MODIS can be observed in this period. Some points, especially in period 1 exceeded the range of estimated uncertainty due likely to ASTER calibration uncertainty that can be underestimated, but most of the points fell within the range of estimated uncertainty in periods 2 and 3.

Figure 4c shows the relative differences between spatially aggregated ASTER band 3N radiances and spectrally adjusted MODIS band 2 radiances as a function of time, which was less than unity for approximately the first half of period 1, resulting in that the slope being statistically significant. The differences were distributed around zero for periods 2 and 3, indicating good agreement between ASTER band 3N and MODIS band 2 (absolute values of $\epsilon_{b}$ were less than 1.0.) The slope is not significant. Most of the points fell within the estimated range of uncertainty.

## 4. Sensitivity Analysis

Reflectances measured by MODIS are SI-traceable to the National Institute of Standards and Technology (NIST) reflectance standards [8], and thus the uncertainty of the cross-calibration of ASTER with reference to MODIS should be estimated in order to ensure error bounds for the cross-calibration results [48,49]. This uncertainty was evaluated according to following equation with two independent variables, namely, uncertainties in spectrally adjusted MODIS radiances and spatially aggregated ASTER radiances:

(5) $$U_{b}^{2}=U_{m,b}^{2}+U_{a,b}^{2}$$

where $U_{b}$ is assumed to be the total uncertainty for the cross-calibration for band b, $U_{m,b}$ is the uncertainty for spectrally adjusted MODIS radiances for band b, and $U_{a,b}$ is the uncertainty for spatially aggregated ASTER radiances for band b. The values of $U_{b}$ were estimated to be 4.93, 4.83, and 5.17 for green, red, and NIR bands, respectively, based on the sensitivity analysis described in the following subsections.

### 4.1. Uncertainty for Spectrally Adjusted MODIS Radiances

Four sources of uncertainty are expected in the spectrally adjusted MODIS radiances: MODIS reflectance calibration uncertainty, errors in atmospheric condition for spectral band adjustment, soil line influences (soil reflectance variability) for spectral band adjustment, and errors in exo-atmospheric solar irradiance model for deriving radiances. The effects of each source on the spectrally adjusted MODIS radiances were estimated based on literature reviews and numerical simulations.

#### 4.1.1. Effects of MODIS Reflectance Calibration Uncertainty

The MODIS calibration uncertainty is reported to be within 2.0% in reflectance units for VNIR bands in the early mission [35]. The calibration for Collection 6 of Terra-MODIS band 1–4 is based on the early mission solar diffuser (SD)/solar diffuser stability monitor (SDSM) and lunar measurements and tracked by the stability of Libya-4 site [50]. Thus, 2.0% uncertainty in the Collection 6 MODIS reflectance calibration was assumed in the present study.

#### 4.1.2. Effects of Errors in Input Parameters for the Radiative Transfer Code for Spectral Band Adjustment

Five parameters of the input of 6SV code, including the AOT at 550 nm, the Junge parameter, the imaginary part of the refractive index, the column water vapor, and the column ozone amount, were perturbed in the sensitivity analysis. The magnitudes of input errors were assumed to be 2σ (two standard deviations) of the measurements in the Railroad Valley Playa shown in Table 1 for four of the parameters (AOT at 550 nm, Junge parameter, column water vapor, and column ozone amount). The value of 2σ is used, because we used a common atmospheric condition when the in-situ atmospheric data are missing and anomalous values of the atmospheric condition could appear even in the clear-sky condition when extreme wind creates blowing dust or the large number of the commercial aircraft create contrails [34]. The common value ±2σ is thus used to perturb the input parameter in the numerical simulation. If the perturbed parameter becomes negative, the minimum value of the atmospheric measurement on the cross-calibration date is used. The refractive index was fixed to be 0.005 in cross-calibration, although, in reality, the index would not be constant and would be affected by aerosol characteristics such as the aerosol size distribution. The imaginary part of the refractive index was thus varied as 0.001, 0.005, and 0.01, respectively.

The ASTER and MODIS radiances with average soil and common atmospheric conditions were computed. Differences between the spectrally adjusted MODIS radiances obtained with perturbed inputs of the atmospheric parameters and the ASTER radiances were computed in order to evaluate the uncertainty due to the effects of atmospheric conditions. This simulation is performed using all possible combinations of perturbed and non-perturbed inputs (common atmospheric condition) for the five parameters, minus the case in which no inputs were perturbed. The standard deviations of the percentage differences between the ASTER radiances and the spectrally adjusted MODIS radiances based on partially/fully perturbed inputs were obtained. The uncertainties were 0.12, 0.14, and 0.81 for the green, red, and NIR bands, respectively. The uncertainties are quite small, especially in the green and red bands. This may be a result of the two-way use of the atmospheric model [33] for the spectral matching band, in which correlations between atmospheric parameters (transmittances, atmospheric reflectances, and spherical albedo) of spectral matching bands of ASTER and MODIS are high so that propagated errors of the input of 6SV are small on spectral band adjustment.

#### 4.1.3. Soil Line Influence on Spectral Band Adjustment

Soil line equations assume perfect overlap of the scatter plot of soil reflectances of spectral matching bands over a single line. This assumption is ideal and introduces uncertainty in the surface reflectance conversion.

The uncertainty was estimated as follows using the 10 datasets of surface reflectances. The ASTER TOA radiances were simulated by 10 soil reflectances and the common atmospheric condition. In addition, 10 soil reflectances were convolved using MODIS RSR in order to provide MODIS band reflectances of the surface, which were then converted to corresponding ASTER bands using the soil line equations. The converted reflectances were ingested into the 6SV code in order to output spectrally adjusted MODIS radiances. The percentage differences between the ASTER radiances and the spectrally adjusted MODIS radiances were computed, and the uncertainty was quantified by 3σ of the relative differences. The reason for using 3σ is that the temporal, spatial, and spectral variability of the soil reflectance of the ROI (1 km × 2 km) would be greater than in the area for in-situ measurements (90 m × 80 m). The averages of standard deviation of radiances for ROI were 2.5–3.6 times greater than that of area for in-situ measurements for VNIR region, which were computed based on data used in cross-calibration. The increase of the standard deviation might be attributed to increasing soil reflectance variability. Also, changes in soil moisture introduce significant changes in spectral reflectances of the surface [34]. Therefore, use of 3σ might be sufficiently large for the sensitivity analysis. The values of uncertainty were 0.61, 0.60, and 1.32 for the green, red, and NIR bands, respectively. The small uncertainties are understandable based on the high values for the coefficient of determination for the regression results of soil reflectances shown in Figure 3 ($R^{2}\;\geq$ 0.997).

#### 4.1.4. Effect of Errors in the Exo-Atmospheric Solar Irradiance Model for Deriving Radiances

The solar irradiance model for VNIR bands provided by the MODIS MCST is a combination of the solar irradiance data of Thuillier et al. [38] and Neckel and Labs [39]. The uncertainty in the irradiance model was estimated based on the results of studies investigating the uncertainty of Thuillier’s solar model over the VNIR and SWIR bands [38,51]. Estimation uncertainty for the green, red, and NIR bands based on 2σ yielded values of 1.96, 1.71, and 2.05 for the green, red, and NIR bands, respectively. 2σ is used because the uncertainty analysis in [51] is based on 2σ. These values are used directly as the uncertainty for the spectrally adjusted MODIS radiances, since radiances can be approximated by a linear function of the solar irradiance values of the band. In a previous study comparing six solar irradiance models [52], ±3% differences were observed in the VNIR bands (>550 nm). The values of uncertainty based on 2σ might therefore be reasonable.

#### 4.1.5. Combined Uncertainty for Spectrally Adjusted MODIS Radiances

The estimated uncertainties in spectrally adjusted MODIS radiances for individual sources of errors are combined to calculate the total uncertainty for each band as shown in Table 4. The root sum of square (RSS) for the uncertainty for each band yielded values of 2.87, 2.70, and 3.26 for the green, red, and NIR bands, respectively. The uncertainty was within the 4% requirement for ASTER calibration [4,6]. The spectral band adjustment would provide uncertainties of 0.62, 0.61, and 1.55 for the green, red, and NIR bands, respectively. The effects of spectral band adjustment were less than half the effects of MODIS calibration uncertainty (approximately 2%) and less than half the effects of solar irradiance in the green and red bands (1.96 and 1.71, respectively). Moreover, the effects of spectral band adjustment were smaller than the effects of solar irradiance in the NIR band (2.05). The total uncertainty in the NIR band was thus slightly greater.

### 4.2. Uncertainty in Spatially Aggregated ASTER Radiances

The uncertainty in spatially aggregated ASTER radiances was computed based on ASTER radiance calibration uncertainty and the uncertainty from geolocation errors of ASTER relative to MODIS. The effects of the relative geolocation errors are included in the uncertainty for ASTER radiances rather than MODIS, since ASTER data were spatially registered to MODIS, i.e., the reference sensor. The absolute accuracy of ASTER radiometric data was assumed to be 4.0% for each band.

The effects of relative geolocation errors were simulated as follows. The absolute geolocation errors for ASTER were reported to be <50 m [53] and those for MODIS were reported to be <45 m [54]. The relative geolocation errors of 100 m for the along- and cross-track directions were assumed to be large enough for our sensitivity analysis. The ASTER data used in the cross-calibration were also used to compute the effects of relative geolocation errors. The center of the ROI of the ASTER image for a specific band and date was shifted in the x and y directions of the UTM coordinates by adding Δx and Δy, respectively, where Δx and Δy are varied as −100, 0, and 100 in order to move the center of ROI in the vertical, horizontal, and diagonal directions (eight directions), respectively. Afterwards, we extracted subset (shifted) images 1 km × 2 km in size for sensitivity analysis. Eight values of spatially aggregated ASTER radiances using the weighting function (Figure 2) were obtained and used to compute a mean of relative differences between the convolved ASTER radiances of non-shifted and shifted data [55]:

(6) $$\ell_{a,i,b}=\frac{1}{8}\frac{\sum_{j=1}^{8}L_{a,b,i,j}\;-\;L_{a,b,i,ROI}}{L_{a,b,i,ROI}}$$

where $\ell_{a,i,b}$ is the mean of relative differences between the ASTER radiances of non-shifted and shifted data for band b and the i-th date. Moreover, $L_{a,b,i,j}$ are the aggregated ASTER radiances for band b, the i-th date, and the j-th direction of shifting. Finally, $L_{a,b,i,ROI}$ are the aggregated ASTER radiances for band b and the i-th date over the ROI. An absolute value of $\ell_{a,i,b}$ was averaged all over dates in order to compute the band-dependent uncertainty, yielding 0.33, 0,33, and 0.30 for the green, red, and NIR bands, respectively. The values of the uncertainty were very small due to the spatial homogeneity of the Railroad Valley Playa.

Table 5 shows the error budget table for spatially aggregated ASTER radiances for ASTER-MODIS cross-calibration of the VNIR bands. The value of the uncertainty was almost band-independent, and the effects of geolocation errors were negligible relative to ASTER radiance calibration uncertainty.

## 5. Derivation of RCCs and Comparison to Independent Calibration Methods

The cross-calibration results were used to derive the RCCs (coefficients for expressing radiometric degradation) on each date, during which the cross-calibration could be performed. The value of the RCC is generally less than unity. The RCC for band b on a date $t_{i}$ based on the cross-calibration (${\mathrm{RCC}}_{cross,b,t_{i}}$) is obtained by the following function:

(7) $${\mathrm{RCC}}_{cross,b,t_{i}}=\frac{{\bar{L}}_{a,b,i}\times {\mathrm{RCC}}_{ver4,b}\left(t_{i}\right)}{{\hat{L}}_{m,b,i}}$$

where ${\mathrm{RCC}}_{ver4,b}$ is the RCC function dependent on a date, and the coefficients for the mathematic function are stored in ASTER radiometric DB version 4. The numerator of Equation (7) corresponds to the aggregated ASTER radiances, in which band-dependent radiometric degradation is not corrected (other corrections such as offset corrections are performed) such that, in general, the radiance values are underestimated relative to radiances without radiometric degradation of the sensor. The ${\mathrm{RCC}}_{cross,b,t_{i}}$ values are compared to the RCC data from an onboard lamp calibration [5] and vicarious calibration using a reflectance-based method [56], which are partially used in the radiometric DB ver. 4. The details of degradation functions for onboard lamp calibration and vicarious calibration and/or cross-calibration are described in Appendix A.

**Figure 5:**
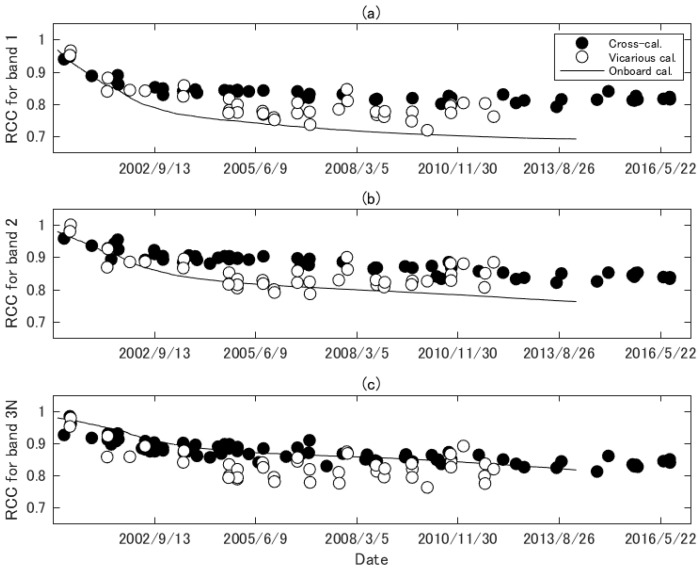
Radiometric calibration coefficients (RCCs) derived by cross-calibration results together with the RCCs provided by onboard calibration and vicarious calibration used in radiometric DB ver. 4. (**a**) ASTER band 1; (**b**) ASTER band 2; and (**c**) ASTER band 3N.

Figure 5 shows ${\mathrm{RCC}}_{cross,b,t_{i}}$ together with the RCC from the onboard and vicarious calibrations over time. The temporal resolution of onboard calibration is much higher than those of the other calibrations, and so the RCC is described by a continuous line. The data for the onboard and vicarious calibrations plotted in Figure 5 were used in the radiometric DB ver. 4, and no data are identified after certain dates, whereas the RCCs of cross-calibration derived in the present study are available until 2016. The variability in the RCCs from cross-calibration was smaller than that from vicarious calibration, indicating that the uncertainty induced by random errors in cross-calibration might have been relatively small for all bands. The RCCs from cross-calibration gradually decreased after launch and exhibited a flat trend after 2008. This trend is similar to that of vicarious calibration for the first five years, but differed between approximately 2005 and 2010. These differences may have been caused by the uncertainty of the cross-calibration and vicarious calibration. The uncertainty in vicarious calibration includes biases in the calibration of a white reference panel for measurements of surface reflectance factor and errors in solar irradiance model data, which are dominant sources of the uncertainty of reflectance-based method [57].

A noticeable difference was found in the relationship between cross-calibration and onboard calibration. The RCC from onboard calibration agreed well with cross-calibration for the first few years, but then began to decrease after the initial period of agreement, as shown in Figure 5a,b. The uncertainty in onboard calibration of ASTER may increase over time, since the harsh environment of space degrades the onboard calibration system and there is no way to correctly monitor the calibration system. The calibration results shown in Figure 5c showed good agreement compared to the results shown in Figure 5a,b.

It is conceivable that the uncertainty of ${\mathrm{RCC}}_{cross,b,t_{i}}$ is approximately equal to that of spectrally adjusted MODIS radiances (2.8 to 3.3%) as provided in the previous section, even if ${\mathrm{RCC}}_{cross,b,t_{i}}$ is influenced by the effects of the errors in ASTER radiometric calibration, except for those due to radiometric degradation. This is because calibration errors other than those due to radiometric degradation will mostly be canceled out when deriving fully calibrated radiances of ASTER using ${\mathrm{RCC}}_{cross,b,t_{i}}$. Note that the derivation of the functions for RCC can suffer from a limited number of data from cross-calibration, leading to a lower value of precision in deriving the RCC functions. The pair of satellite sensors that collect data more frequently, such as Landsat 7 Enhanced Thematic Mapper Plus (ETM+) and Terra MODIS, would, however, provide a greater number of datasets for cross-calibration, as well as more precise and statistically significant results of the cross-calibration [58]. Further discussion is necessary in order to select or integrate RCCs from individual calibration methods relying on bands, which is beyond the scope of the present study.

## 6. Discussion

Given that the absolute and relative (inter-band) radiometric accuracy of MODIS bands 4, 1, and 2 is sufficiently high, our cross-calibration results indicate that radiometric measurements of ASTER bands 1 and 2 are larger than those of band 3N. Such a relationship between ASTER VNIR bands is consistent with the results for inter-band radiometric compassion/calibration of ASTER VNIR bands [9]. A similar trend was also observed in the previous study of the ASTER-MODIS cross-calibration [14]. However, ASTER band 3N was 5% smaller than that of the corresponding MODIS band, while a bias of <1% was found between ASTER and MODIS NIR bands in the present study. The differences in the results for the NIR band are due primarily to the uncertainty in spectral band adjustment. Spectral band adjustments for the present study are based on historical data of surface reflectance and the radiative transfer model, whereas the previous study relied on hyperspectral data from EO-1 Hyperion. Further investigation is required in order to identify the source of these differences.

The present study used a model-based spectral band adjustment rather than using the SBAF calculated from hyperspectral sensors, since the sensitivity analysis provided the possible range of uncertainty in order to clarify the results of cross-calibration (the uncertainty of the model-based spectral adjustment was 0.6% for bands 1 and 2 and 1.6% for band 3N as shown in Table 4). At present, this approach is limited to the Railroad Valley Playa and cannot be applied to other calibration sites, such as pseudo-invariant calibration sites (PICS), because historical data of surface reflectance of the site are not available. In order to conduct cross-calibration using multiple sites such as PICS, we may need the SBAF derived using hyperspectral sensors. In such a case, the uncertainty and feasibility of the SBAF for the ASTER-MODIS cross-calibration should be fully investigated.

The calibration uncertainty of MODIS and errors in the solar irradiance model for the derivation of radiances were identified as the two major sources of uncertainty in the reference radiances. In the NIR band, errors in spectral band adjustment also degrade the accuracy of cross-calibration. Note that the effects of errors in the solar irradiance model on the ASTER-MODIS cross-calibration are not negligible [59], but improved accuracy in the solar irradiance model contributes to improvement in the accuracy of the cross-calibration.

The study site used in the present study was limited to a single site. Further systematic studies will be needed to cover various types of land surfaces.

Having cross-calibrated data from two sensors, which are radiometrically consistent with each other, might be a prerequisite for improving accuracy in the synergistic application of data from multiple sensors. It is hoped that the results of the present study will help to improve accuracy in application research using combined MODIS and ASTER data, such as biomass estimation [60], investigation of the structure and ecological functioning of urban systems [61], 3-D cloud radiative interaction research [62], and inter-comparison of reflectance and vegetation indices for use in synergistic applications [63,64].

## 7. Conclusions

The cross-calibration results indicated that ASTER bands 1 and 2 are on averages 3.9% and 3.4% greater than MODIS bands 4 and 1 and ASTER band 3N (nadir) is 0.6% smaller than MODIS band 2. %RMSEs between radiances of two sensors were 3.7, 4.2, and 2.3 for bands 1, 2, and 3N, respectively, which are slightly greater or smaller than the 4% requirement of ASTER radiometric calibration. Numerous points in the calibration results (relative errors between spatially aggregated ASTER and spectrally adjusted MODIS radiances) fell within the estimated range of uncertainty (4.8 to 5.2%), but some points exceeded the range of the estimated uncertainty due likely to underestimation of uncertainties and/or unaccounted errors. The uncertainties in the cross-calibration were induced mainly by the calibration uncertainties of ASTER radiances and MODIS reflectances, as well as errors of the exo-atmospheric solar irradiance model used to derive radiances. The RCCs based on the cross-calibration results were derived in order to improve ASTER radiometric calibration. In addition, the uncertainty in the RCCs (2.8 to 3.3%) was identified to be within the 4% radiometric accuracy requirement of ASTER. Therefore, using these RCCs might be acceptable for ASTER radiometric calibration.

The use of the derived RCCs based on the improved cross-calibration algorithm can reduce errors of radiometric calibration of ASTER for bands 1 and 2, calibrated by combined vicarious calibration and cross-calibration. This can be achieved by replacing the set of cross-calibration-based RCCs for the current radiometric DB with the RCCs obtained in the present study in order to derive the RCC curves. The derived RCC for band 3N ensures good agreement with the current RCC functions, which are calibrated primarily by onboard calibration. The improved radiometric consistency, i.e., interoperability between ASTER and MODIS will improve analysis accuracy for synergistic applications using these two sensors.

## Figures and Tables

**Table 1:** Median, mean, and standard deviation (STD) of atmospheric parameters calculated using measurements from AERONET and TOMS/OMI for the Railroad Valley Playa.

	AOT at 550 nm	Junge Parameter	Water Vapor (g/cm^2^)	Ozone (DU)
Median	0.045	3.23	0.73	294.0
Mean	0.058	3.18	0.79	296.7
STD	0.034	0.53	0.41	27.8

**Table 2:** Values of $\epsilon_{b}$ and ${\%\mathrm{RMSE}}_{b}$ for the entire period.

	A1, M4	A2, M1	A3N, M2
$\epsilon_{b}$	3.92	3.64	−0.61
${\%\mathrm{RMSE}}_{b}$	3.65	4.20	2.32

**Table 3:** Values of $\epsilon_{b}$ and ${\%\mathrm{RMSE}}_{b}$ for periods 1, 2, and 3, the slopes of the regression lines for the relative differences between spatially aggregated ASTER radiances and spectrally adjusted MODIS radiances as a function of time, F-statistics obtained from the F-test of the regression model, and *p*-values for each period.

		Period 1	Period 2	Period 3
$\epsilon_{b}$	A1, M4	3.84	4.28	3.74
	A2, M1	5.65	3.41	−0.10
	A3N, M2	−1.61	0.20	0.91
${\%\mathrm{RMSE}}_{b}$	A1, M4	3.73	3.59	3.66
	A2, M1	5.45	3.27	1.23
	A3N, M2	2.78	1.79	1.47
Slope	A1, M4	3.35 × 10^−5^	−8.95 × 10^−6^	5.01 × 10^−6^
	A2, M1	5.38 × 10^−6^	−2.06 × 10^−5^	1.62 × 10^−6^
	A3N, M2	2.08 × 10^−5^	5.47 × 10^−7^	1.00 × 10^−5^
F-value	A1, M4	71.9	2.14	0.497
	A2, M1	1.03	8.39	0.0692
	A3N, M2	15.5	0.00511	1.98
*p*-value	A1, M4	1.63 × 10^−7^	0.178	0.494
	A2, M1	0.32	0.0117	0.797
	A3N, M2	3.52 × 10^−4^	0.944	0.185

**Table 4:** Error budget table for spectrally adjusted MODIS (reference) radiances for ASTER-MODIS cross-calibration of VNIR bands showing individual uncertainty factors and combined uncertainty. The three right-most columns list the uncertainty in spectrally adjusted MODIS radiances. In the table, spectral band adjustment is abbreviated as SBA.

No.	Name of Source		Source Error	Green	Red	NIR
1	MODIS reflectance calibration uncertainty		-	2.0		
2	Atmospheric condition for SBA	AOT at 550 nm	0.068 (2σ)	0.12	0.14	0.81
		Junge parameter	1.06 (2σ)			
		Refractive index (imaginary part)	100%			
		Water vapor amount	0.82 (/cm^2^) (2σ)			
		Ozone column content	55.6 (DU) (2σ)			
3	Soil line influence for SBA		-	0.61	0.60	1.32
4	Solar irradiance error for deriving radiances		2σ	1.96	1.71	2.05
	RSS for SBA (Nos. 2–3)			0.62	0.61	1.55
	RSS (Nos. 1–4): $U_{m,b}$			2.87	2.70	3.26

**Table 5:** Error budget table for spatially aggregated ASTER radiances for ASTER-MODIS cross-calibration VNIR bands showing individual uncertainty factor and combined uncertainty. The three right-most columns list the uncertainty in spatially aggregated ASTER radiances.

No.	Source of Uncertainty	Source Error	Green	Red	NIR
1	ASTER radiance calibration uncertainty	4.0%	4.0		
2	Geolocation errors relative to MODIS	100 m	0.33	0.33	0.3
	RSS: $U_{a,b}$		4.01	4.01	4.01

## Appendix A

The model for radiometric degradation based on onboard calibration can be represented by following form [4]:

(A1) $${\mathrm{RCC}}_{onb,b}\left(t_{i}\right)=\{\begin{array}{c} \;c_{onb1,0,b,T}\;+\;c_{onb1,1,b,T}\;\times \;t_{i}\;+\;c_{onb1,2,b,T}\;\times \;t_{i}^{2}\;\;\mathrm{for}\;t_{i}\;<\;672\\ c_{onb2,0,b,T}\exp\left(-c_{onb2,1,b,T}\;\times \;t_{i}\right)\;+\;c_{onb2,2,b,T}\;\;\mathrm{for}\;t_{i}\;>\;672 \end{array}$$

where $t_{i}$ is a date since the launch, ${\mathrm{RCC}}_{onb,b}$ is RCC for onboard calibration for band b and a subscript “onb” indicates onboard calibration. $c_{onb1,k,b,T}$ indicates the coefficients for the model for $t_{i}\;<\;672$, a subscript k corresponds to 0, 1, or 2, respectively, b represents band, and T identifies the period of the function (T=1, 2, …, 15), meaning that the coefficients depend on the period out of fifteen. $c_{onb2,k,b,T}$ indicates the coefficients for the model for $t_{i}\;>\;672$. At present, this function is used for ASTER band 3N. The onboard calibration lines in Figure 5 are derived by Equation (A1). Notice that the calibration coefficient after the 4876th day since launch is set to a constant based on the results of vicarious calibration [9].

The model for radiometric degradation based on vicarious calibration and/or cross-calibration can be represented by following form [56]:

(A2) $${\mathrm{RCC}}_{cal,b}\left(t_{i}\right)=c_{cal,0,b}\left[1.0\;-\;c_{cal,1,b}\right]\exp\left(-c_{cal,2,b}\;\times \;t_{i}\right)+\;c_{cal,0,b}\;\times \;c_{cal,1,b}$$

where ${\mathrm{RCC}}_{cal,b}$ is RCC for vicarious calibration and/or cross-calibration for band b and a subscript “cal” identifies calibration method (vicarious calibration and/or cross-calibration). $c_{cal,k,b}$ indicates the coefficients of the model for band b, and k corresponds 0, 1, or 2, respectively. This function is currently used to calibrate ASTER bands 1 and 2. The curves based on Equation (A2) are not described in Figure 5.
