# Correction: Obata, K., et al. Cross-Calibration between ASTER and MODIS Visible to Near-Infrared Bands for Improvement of ASTER Radiometric Calibration. *Sensors* 2017, *17*, 1793

**DOI:** 10.3390/s20144057

**Published:** 2020-07-21

**Authors:** Kenta Obata, Satoshi Tsuchida, Hirokazu Yamamoto, Kurt Thome

**Affiliations:** 1 National Institute of Advanced Industrial Science and Technology (AIST), The Institute of Geology and Geoinformation, Tsukuba, Ibaraki 305-8567, Japan; s.tsuchida@aist.go.jp (S.T.); hirokazu.yamamoto@aist.go.jp (H.Y.); 2 Department of Information Science and Technology, Aichi Prefectural University, Nagakute, Aichi 480-1198, Japan; 3 NASA Goddard Space Flight Center, Greenbelt, MD 20771, USA; kurtis.thome@nasa.gov

The authors wish to make the following corrections to this paper [1]:

On page 12, there is an error in Equation (6) and the related text. 

The original sentences are as follows: 

“Eight values of spatially aggregated ASTER radiances using the weighting function (Figure 2) were obtained and used to compute a mean of relative differences between the convolved ASTER radiances of non-shifted and shifted data [55]:

(6) $$\ell_{a,i,b}=\frac{1}{8}\frac{\sum_{j=1}^{8}L_{a,b,i,j}-L_{a,b,i,ROI}}{L_{a,b,i,ROI}}$$

where $\ell_{a,i,b}$ is the mean of relative differences between the ASTER radiances of non-shifted and shifted data for band b and the i–th date. Moreover, $L_{a,b,i,j}$ are the aggregated ASTER radiances for band b, the i–th date, and the j-th direction of shifting. Finally, $L_{a,b,i,ROI}$ are the aggregated ASTER radiances for band b and the i–th date over the ROI. An absolute value of $\ell_{a,i,b}$ was averaged all over dates in order to compute the band-dependent uncertainty, yielding 0.33, 0,33, and 0.30 for the green, red, and NIR bands, respectively.”

In Equation (6), the symbol for absolute value should be applied to the term “$L_{a,b,i,j}-L_{a,b,i,ROI}$”. The wording of the sentences related to the equation should be modified, with “mean of relative differences” corrected to “relative value of the mean absolute difference”. The sentence regarding the computation of the average of $\ell_{a,i,b}$ should be modified.

The authors confirm that the numerical experiments reported in the paper were conducted properly, and that the above changes do not affect the results. The authors apologize for any inconvenience caused by these changes. 
